# Epidemiologic Associations Between Inflammatory Bowel Disease and Hodgkin Lymphoma or Multiple Sclerosis

**DOI:** 10.1016/j.gastha.2024.03.013

**Published:** 2024-03-26

**Authors:** Amnon Sonnenberg, Ruth Kohen

**Affiliations:** 1 Section of Gastroenterology, Portland VA Medical Center, Portland, Oregon; 2 Division of Gastroenterology and Hepatology, Oregon Health & Science University, Portland, Oregon; 3 Department of Psychiatry, University of Washington, Seattle, Washington

**Keywords:** Environmental Risk Factors, Epidemiology of IBD, Etiology of IBD, Epstein-Barr Virus, Medicare Population

## Abstract

**Background and Aims:**

Epidemiologic evidence suggests that Hodgkin lymphoma (HL) and multiple sclerosis (MS) share a common set of risk factors with Crohn’s disease (CD) and ulcerative colitis (UC). It was hypothesized that such shared risk factors would lead to similar geographic distributions of these 4 diagnoses and their concurrence in identical patients.

**Methods:**

All subjects with HL, MS, CD, or UC were identified in the complete Inpatient Standard Analytic File of the Centers for Medicare and Medicaid Services from 2018. In a cross-sectional study, we evaluated whether the frequencies of HL, MS, CD, and UC occurrences among different US states were statistically correlated with each other. In a case-control study, the observed concurrences of each 2 of the 4 diagnoses were compared with their expected frequencies in the overall Medicare population by calculating odds ratios with their 95% confidence intervals.

**Results:**

The total Centers for Medicare and Medicaid Services population comprised 6,462,321 unique patients, of whom 8027 presented with HL, 42,934 with MS, 40,623 with CD, and 32,521 with UC. Statistically significant positive correlations (r) with *P* < .001 were found between HL and MS (r = 0.50), HL and CD (0.46), HL and UC (0.68), MS and CD (0.66), MS and UC (0.72), and CD and UC (0.68). Any inflammatory bowel disease was significantly associated with a diagnosis of concurrent HL (odds ratio: 1.22, 95% confidence interval: 1.01–1.48) or MS (1.35, 1.25–1.46).

**Conclusion:**

The epidemiologic associations of inflammatory bowel disease with HL or MS may reflect a common pathway in the etiology or pathogenesis of these diseases.

## Graphical abstract

**Figure:**
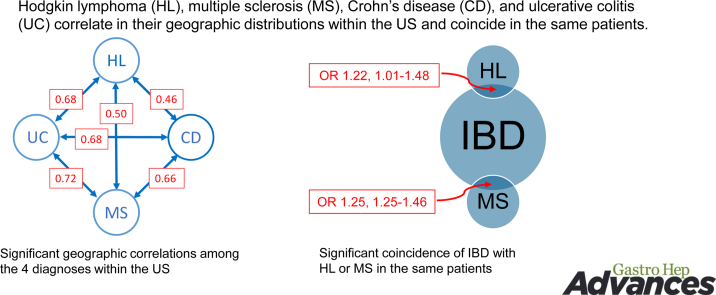

## Introduction

The causation of both types of inflammatory bowel disease (IBD), that is, Crohn’s disease (CD) and ulcerative colitis (UC), is still unknown. The study of their epidemiology serves to reveal patterns of disease variation with respect to time, geography, or different demographic groups. It is hoped that eventually such epidemiologic patterns will provide clues about potential environmental risk factors that influence the occurrence of IBD. A particular risk factor could be revealed if it varied by geography, time, or demographics in a similar fashion as IBD itself. In general, large patient populations are needed to study the epidemiologic variations associated with relatively rare diagnoses, such as CD and UC.

The Epstein-Barr virus (EBV) is known to play a role in the occurrence of Hodgkin lymphoma (HL) and multiple sclerosis (MS).^1^, ^2^, ^3^ Several previous epidemiologic studies have shown similar epidemiologic variations of HL, MS, CD, and UC, which could suggest that infection with EBV also contributes to the etiology of IBD. A previous meta-analysis showed that patients with CD or UC harbor a 1.5-fold increased risk for concurrent MS.^4^ Case series and population-based cross-sectional studies suggested an increased risk for HL in patients with CD and UC.^5^^,^^6^ A case-control study in the US veteran population suggested that all 4 diagnoses tend to significantly coincide in identical patients.^7^ The long-term time trends of HL, MS, CD, and UC are characterized by strikingly similar temporal variations.^8^^,^^9^ Lastly, mortality data for the 4 diagnoses also revealed their similar geographic distributions within the United States as well as among different countries across the globe.^10^^,^^11^

We hypothesized that an epidemiologic study of associations among HL, MS, CD, and UC in the US Medicare population would confirm the previously observed relationships among the 4 diagnoses. The aim of the present study was to utilize the database of the Centers for Medicare and Medicaid Services (CMS) for the analysis of the geographic distributions of these 4 diagnoses across the US and test whether these diagnoses concur in identical patients.

## Methods

The study utilized the Inpatient Standard Analytic File of the CMS of the year 2018. The dataset from 2018 represented the most recent annual datafile from CMS that was still unaffected by the subsequent COVID-19 epidemic. The data resides in the public domain and can be requested through the CMS website. The data of individual patients were deidentified, and all analyses dealt with aggregate data only. For these reasons, the studies were exempt from the need to obtain informed consent from individual patients or approval by the institutional review board.

The present study included all inpatient records within the 2018 data file. In addition to an admitting diagnosis and a principal diagnosis, each record could contain up to 25 diagnoses. De-identified participant numbers were used to aggregate multiple records belonging to the same patient into a single entry used for analysis. Aggregate participant entries used the demographic information pertaining to their first available record and all diagnoses accumulated during multiple inpatient encounters. The occurrence of any diagnosis was determined based on its coding according to the 10th revision of the International Classification of Diseases (ICD10). Patients with CD or UC were identified based on their corresponding ICD10 codes K50 and K51, respectively. Patients with HL were identified based on the ICD10 codes C81 or Z85.71; patients with MS were identified based on the ICD code G35. No further 4-letter subcodes or any other diseases were considered in the present analysis. Besides the ICD10 codes, we also extracted demographic data pertaining to the patients’ ethnicity (White, Black, Hispanic, Asian, or others), sex, and age. No data pertaining to medications were available in the data file.

In a cross-sectional study, we evaluated whether the relative frequency of HL, MS, CD, and UC occurrence among different states were statistically correlated with each other. Frequency of occurrence was calculated as the prevalence of patients with HL, MS, CD, or UC per 10,000 of the total population of all inpatients from the same state and year. Using linear regression analysis, we calculated 6 Pearson’s correlation coefficients (r) between the geographic distributions of each 2 of the 4 diagnoses of interest. We also used weighted regression analysis to adjust for the different population sizes in different states.

In a case-control study, we evaluated whether the *observed* concurrences of HL, MS, CD, and UC were statistically different from the *expected* concurrences based on the overall frequency of each individual diagnosis in the total Medicare population of the same year. For univariate comparisons of disease frequency alone or concurrently with another disease, we calculated odds ratios with their 95% confidence intervals. The concurrence of any 2 diagnoses was considered statistically significant, if the 95% confidence interval of the corresponding odds ratio did not include unity. Differences in the demographic characteristics (age, sex, and ethnicity) between case and control subjects were assessed using t-tests or chi-square analysis. We also used multivariate logistic regressions to adjust the odds ratios for the potential confounding influences of demographic characteristics (age, sex, and race/ethnicity).

## Results

The Medicare data file of 2018 contained 10,982,347 records of 6,462,321 unique patients, of whom 40,623 presented with CD, 32,521 with UC, 8027 with HL, and 42,934 with MS. The comparison (control) population of Medicare patients comprised 6,340,437 unique patients without any of the 4 diagnoses. Table 1 contains additional stratifications of the data by age, sex, and ethnicity. Patients with either MS or CD tended to be younger than the control population (*P* < .001). The patient populations with CD, UC, and MS comprised more females, whereas the populations with HL comprised fewer females than the comparison population (*P* < .001). Lastly, the fraction of Caucasians was slightly higher in all 4 disease groups than in the control population, the difference being more striking in IBD than HL or MS (*P* < .01).

**Table 1:** Medicare Population of 2018 Stratified by Diagnosis, Ethnicity, Sex, and Age

Demographic characteristic	Controls		Crohn's disease		Ulcerative colitis		Hodgkin lymphoma		Multiple sclerosis	
	N	(%)	N	(%)	N	(%)	N	(%)	N	(%)
Total	6,340,437	(100.0)	40,623	(100.0)	32,521	(100.0)	8027	(100.0)	42,934	(100.0)
Age										
Mean age (SD)	74.7	(8.6)	71.2	(8.1)	73.9	(8.3)	72.1	(8.1)	68.0	(6.5)
Gender										
Female	3,409,526	(53.8)	24,462	(60.2)	18,585	(57.1)	3747	(46.7)	30,909	(72.0)
Male	2,930,911	(46.2)	16,161	(39.8)	13,936	(42.9)	4280	(53.3)	12,025	(28.0)
Ethnicity										
White	5,208,279	(82.1)	35,606	(87.6)	28,474	(87.6)	6802	(84.7)	35,516	(82.7)
Black	694,944	(11.0)	3247	(8.0)	2279	(7.0)	710	(8.8)	5503	(12.8)
Asian	92,415	(1.5)	173	(0.4)	321	(1.0)	60	(0.7)	158	(0.4)
Hispanic	132,391	(2.1)	393	(1.0)	460	(1.4)	159	(2.0)	664	(1.5)
Other	167,051	(2.7)	1008	(2.5)	841	(2.6)	272	(3.4)	893	(2.0)

Table 2 contains the 4 disease populations and the total Medicare population stratified by state of residence. A group of 6003 patients resided outside the 50 states of the Union, Puerto Rico, or the District of Columbia. MS had the highest frequency of occurrence, followed by CD and UC. HL was the least frequent of the 4 diagnoses. For each diagnosis, the frequency rates of occurrence varied between 2.9 and 8.6-fold among different states, with HL and CD showing the smallest and largest state-related variation, respectively. The geographic distributions of disease prevalence were characterized by a slight north-south gradient, however, with *multiple* exceptions to the rule. For instance, several of the northern states (Massachusetts, Michigan, New Hampshire, Oregon, Washington, and Wisconsin) were characterized by relatively higher rates, whereas several of the southern states (Alabama, Arizona, Arkansas, Georgia, Kentucky, and Tennessee) were characterized by relatively lower rates. Using the entire dataset of all 50 states, Puerto Rico, and the District of Columbia, statistically significant positive correlations (with *P* < .01) were found between HL and MS (r = 0.36), HL and CD (0.46), HL and UC (0.58), MS and CD (0.66), MS and UC (0.69), CD and UC (0.68). The regression analyses including HL were partly compromised by the low patient counts in the smaller states. Restricting the analysis to 35 states with the largest populations slightly improved the 6 correlation coefficients among the 4 diagnoses (Figure). Using a weighted regression analysis did not further improve the correlation coefficients.

**Table 2:** Patient Counts and Frequency Rates of HL, MS, CD, and UC in Different States

State	Patient counts					Rate per 10,000 POP			
	HL	MS	CD	UC	POP	HL	MS	CD	UC
Alabama	121	565	695	492	118,283	9.54	69.14	52.45	46.88
Alaska	12	87	66	59	12,584	13.36	57.73	56.28	53.46
Arizona	147	635	619	588	109,994	10.52	48.87	53.89	38.69
Arkansas	90	418	461	331	85,541	11.90	56.25	47.06	50.45
California	591	2793	2337	2505	496,551	11.86	96.83	55.88	47.46
Colorado	93	759	438	372	78,383	18.65	78.59	81.75	65.97
Connecticut	136	573	596	481	72,908	14.44	79.41	51.56	53.97
Delaware	42	231	150	157	29,091	11.68	69.30	42.83	34.26
District of Columbia	15	89	55	44	12,842	10.82	55.98	61.34	50.87
Florida	493	2551	2795	2318	455,685	10.48	50.90	61.94	42.07
Georgia	184	894	1088	739	175,655	12.08	20.13	28.86	26.17
Hawaii	18	30	43	39	14,900	14.04	70.85	71.83	43.75
Idaho	43	217	220	134	30,630	12.59	74.78	64.07	51.72
Illinois	368	2186	1873	1512	292,335	10.60	69.47	75.89	48.65
Indiana	165	1081	1181	757	155,613	13.00	58.69	59.06	45.69
Iowa	105	474	477	369	80,760	9.76	64.17	69.12	50.85
Kansas	77	506	545	401	78,854	9.70	57.85	70.38	38.54
Kentucky	113	674	820	449	116,506	10.09	41.12	50.81	32.19
Louisiana	104	424	524	332	103,122	14.00	81.93	104.57	52.43
Maine	47	275	351	176	33,567	13.59	72.34	73.60	54.90
Maryland	194	1033	1051	784	142,796	15.85	84.44	88.62	69.51
Massachusetts	273	1454	1526	1197	172,199	11.84	98.44	74.05	60.36
Michigan	269	2236	1682	1371	227,146	15.56	81.58	66.09	54.67
Minnesota	203	1064	862	713	130,424	8.13	45.26	42.18	31.86
Mississippi	74	412	384	290	91,034	11.85	70.18	67.24	47.04
Missouri	173	1025	982	687	146,046	13.62	80.61	59.42	40.87
Montana	36	213	157	108	26,424	11.73	74.91	65.65	51.86
Nebraska	57	364	319	252	48,594	14.32	88.47	56.14	51.69
Nevada	74	457	290	267	51,658	14.90	75.53	89.64	51.48
New Hampshire	57	289	343	197	38,265	14.92	74.76	58.40	67.58
New Jersey	301	1508	1178	1363	201,701	8.74	51.92	40.17	39.62
New Mexico	32	190	147	145	36,594	15.41	73.56	65.09	64.27
New York	549	2621	2319	2290	356,284	11.61	59.80	61.17	43.92
North Carolina	245	1262	1291	927	211,044	14.24	62.56	61.03	52.90
North Dakota	28	123	120	104	19,661	13.96	73.11	73.80	49.54
Ohio	340	1781	1798	1207	243,618	7.74	50.55	60.53	36.09
Oklahoma	83	542	649	387	107,226	15.47	81.58	65.46	45.43
Oregon	95	501	402	279	61,414	15.60	82.25	68.51	63.58
Pennsylvania	427	2251	1875	1740	273,684	9.81	15.01	12.12	32.90
Puerto Rico	17	26	21	57	17,323	22.60	62.56	90.94	64.13
Rhode Island	43	119	173	122	19,023	11.22	48.49	57.37	36.77
South Carolina	134	579	685	439	119,399	9.66	69.57	68.41	53.72
South Dakota	25	180	177	139	25,873	11.12	56.36	62.16	44.40
Tennessee	159	806	889	635	143,009	10.78	52.38	48.87	42.11
Texas	467	2269	2117	1824	433,171	8.98	89.21	63.14	43.73
Utah	31	308	218	151	34,527	8.11	83.27	72.46	62.19
Vermont	15	154	134	115	18,493	11.68	17.51	23.35	17.51
Virginia	252	1236	1259	929	194,208	12.06	83.52	67.63	44.87
Washington	151	1046	847	562	125,238	11.28	51.39	75.70	48.09
West Virginia	65	296	436	277	57,599	11.28	51.39	75.70	48.09
Wisconsin	169	976	862	626	113,313	14.91	86.13	76.07	55.25
Wyoming	17	142	83	72	15,526	10.95	91.46	53.46	46.37

POP, entire Medicare population.

**Figure:**
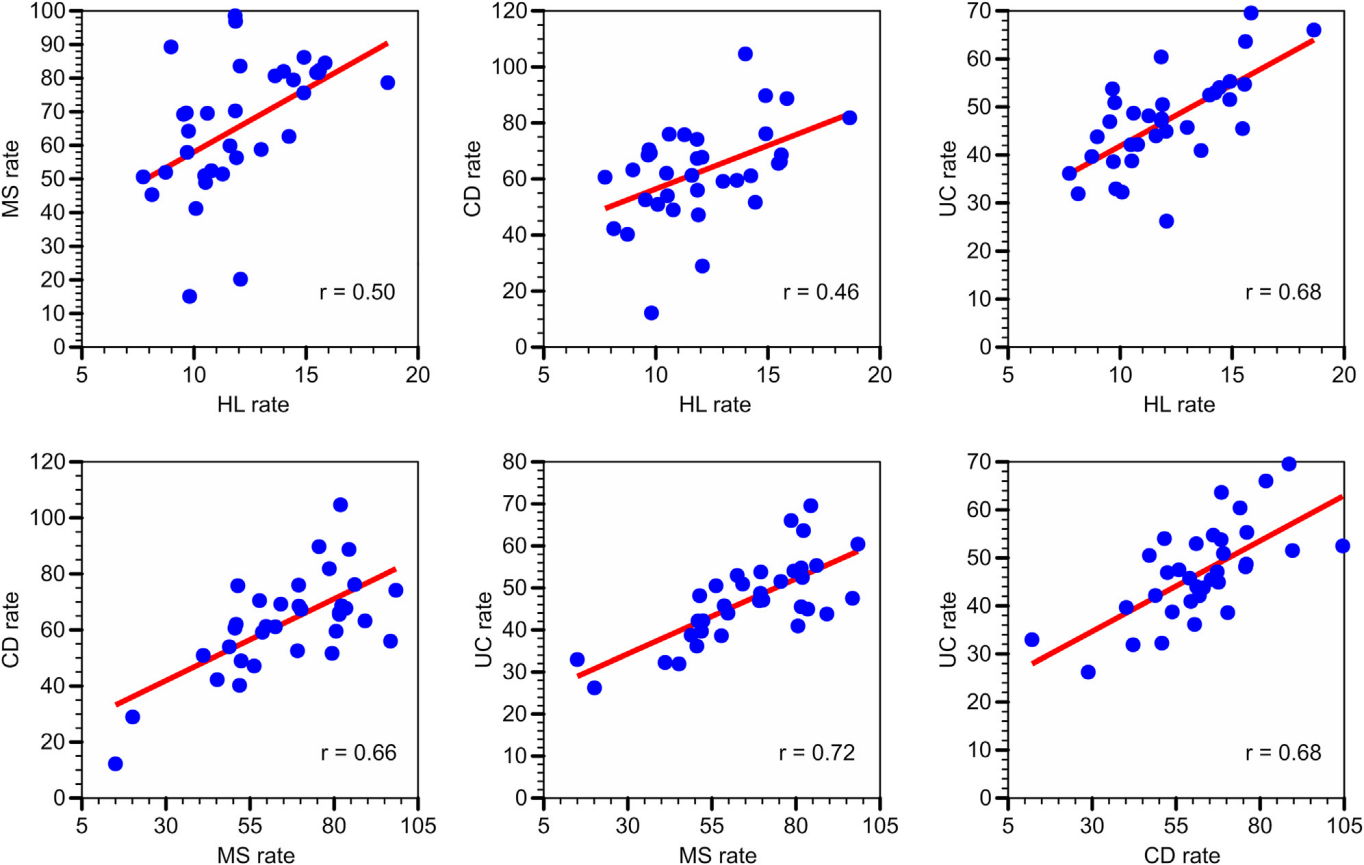
Correlations in the geographic distributions of Hodgkin lymphoma (HL), multiple sclerosis (MS), Crohn’s disease (CD), and ulcerative colitis (UC) among the 35 largest US states. Each data point represents a different state. Rates are expressed per 10,000 patients in the entire Medicare population.

Table 3 lists the concurrences of IBD with HL or MS. A diagnosis of CD was significantly more frequently associated with a concurrent diagnosis of HL or MS. A diagnosis of UC was significantly associated with a concurrent diagnosis of MS, but not HL. Overall, the diagnosis of any IBD was significantly associated with a concurrent diagnosis of HL or MS. No further improvement could be achieved by adjusting the odds ratios to the demographic characteristics.

**Table 3:** Concurrence of Hodgkin Lymphoma (HL) or Multiple Sclerosis (MS) With Crohn’s Disease (CD) or Ulcerative Colitis (UC) in the Medicare Population

1^st^ Diagnosis	2^nd^ Diagnosis	Control	Odds ratio	95% CI
Crohn's disease vs Hodgkin lymphoma				
	HL	none		
CD	65	40,558	1.29	(1.01–1.65)
none	7962	6,413,736		
Crohn's disease vs multiple sclerosis				
	MS	none		
CD	346	42,588	1.29	(1.16–1.43)
none	40,277	6,379,110		
Ulcerative colitis vs Hodgkin lymphoma				
	HL	none		
UC	44	32,477	1.09	(0.81–1.47)
none	7983	6,421,817		
Ulcerative colitis vs multiple sclerosis				
	MS	none		
UC	308	42,626	1.43	(1.28–1.60)
none	32,213	6,387,174		
IBD vs Hodgkin lymphoma				
	HL	none		
IBD	108	71,599	1.22	(1.01–1.48)
none	7919	6,382,695		
IBD vs multiple sclerosis				
	MS	none		
IBD	641	71,066	1.35	(1.25–1.46)
none	42,293	6,348,321		

CI, confidence interval.

## Discussion

Using the electronic dataset of the entire Medicare inpatient population from 2018, the present epidemiologic analysis focused on potential associations between IBD and HL or MS. The investigators hypothesized that HL, MS, CD, and UC would be characterized by similar geographic variations within the US and that these 4 diagnoses would also tend to coincide more frequently in identical patients. Both hypotheses were confirmed by the results of the present analyses. In previous studies, the analysis of the geographic distribution was based solely on mortality data.^10^^,^^11^ The present analysis reveals that the similarity in the geographic distributions of the 4 diagnoses is found in other types of healthcare statistics as well. The coincidence of the 4 diagnoses in identical patients was initially observed in the US veteran population but had not yet been confirmed in other datasets of current healthcare records.^7^ Overall, the present results lend additional support to the hypothesis of a shared environmental risk factor or a common pathway in the pathogenesis of these 4 distinct diagnoses.

The long-term time trends of HL, MS, CD, and UC are shaped by strikingly similar birth-cohort patterns.^8^^,^^9^ Any type of birth-cohort pattern is highly suggestive of exposure to an environmental risk factor during early lifetime with long-lasting medical consequences. The risk factor itself or its lasting impact on pathophysiology can then affect the exposed subject’s susceptibility to develop the disease many years after the initial exposure. Such patterns are frequently associated with bacterial or viral infections during childhood or early adulthood. In gastroenterology, the acquisition of *Helicobacter pylori* infection during childhood and the subsequent development of peptic ulcer or gastric cancer decades later represent a typical example for such behavior.^12^ The association of both HL and MS with a prior EBV infection makes one wonder whether EBV might also play a role in the etiology of CD and UC.^1^, ^2^, ^3^ A recent study of mortality from these 4 diagnoses in different countries revealed similar geographic patterns and significant correlations among their world-wide distributions.^10^ This study also suggested that the exposure to any relevant environmental risk factors must have started before the age of 5 years in UC and HL and before the age of 15 years in CD and MS.

The Medicare population is limited by its restriction to patients older than 65 years, whereas HL, MS, CD, and UC tend to mostly affect patients during early or mid-adult life. Previous diagnoses may have gone unnoticed or unrecorded during the inpatient encounter that entered the Medicare datafile. Our analysis was restricted to a single year, which did not provide the opportunity to study the sequence of disease occurrence in individual patients. Future studies, involving a younger patient population with follow-ups over prolonged time periods, may provide the opportunity to study such sequential disease occurrences in individual patients.

The analysis was also limited by the absence of additional information about social habits or types of immune-modulating therapy. Theoretically, the epidemiologic associations between IBD with HL or MS could relate to adverse effects of immune suppressive therapy.^13^^,^^14^ As the mechanism remains unknown, by which immune-suppressive therapy would lead to an increased risk of HL or MS, it is conceivable that such a mechanism also involves reactivation of dormant EBV infection. The shortcomings in trying to record the usage of immune-suppressive medications as a confounding variable relate to difficulties in retrieving reliable information about the length of time and overall amount of exposure to a large variety of such medications for each individual case subject, let alone the entire control population. Most importantly, the strong association between IBD diagnosis and immune-suppressive therapy also limits the usefulness of any information about medications as an independent co-variable. Although the use of immune-suppressive medications for IBD treatment has markedly increased during the past 3 decades, there is no indication that the concurrence of HL or MS with IBD has increased likewise.^7^ It would be difficult to explain the similar birth-cohort patterns or geographic distributions of the 4 diagnoses based on the effects of immune suppressive therapy.

Despite their statistical significance, the odds ratios for the associations between IBD and HL or UC are relatively weak. The epidemiologic data already indicate that exposure at different ages may underlie the occurrence of the 4 different diagnoses.^9^^,^^10^ Exposure at different ages and different mechanisms of pathophysiology may result in different phenotypes associated with exposure to the same agent. Although over 90% of the adult population harbors antibodies against EBV, only a minute fraction ever develops HL or multiple sclerosis. Referring again to the example of *H. pylori*, only a small fraction of all infected subjects develop peptic ulcers, and an even smaller fraction of ulcer patients subsequently go on to develop gastric cancer.^12^

At the present time, the nature of the environmental risk factors affecting the epidemiologic associations between IBD and HL or MS remains speculative. Besides EBV infection, other risk factors may contribute to the observed epidemiologic patterns. The north-south gradient in the occurrence of MS and IBD has led previous investigators to speculate that increased sun exposure with high vitamin D serum levels provides a protective influence with respect to IBD and MS.^15^^,^^16^ In general, cold climate may force people to spend more time indoors and expose themselves to rampant infections. The occurrences of MS, HL, and IBD have also been associated with Caucasian ethnicity and economic affluence.^17^, ^18^, ^19^, ^20^ As much as these epidemiologic parameters vary in their magnitude across the United States, they may also have contributed to the occurrence of similar geographic variations of MS, HL, and IBD.^21^^,^^22^

## Conclusion

Using the electronic database of the US Medicare population, the present analysis revealed significant correlations between the geographic distribution of HL, MS, CD, and UC across the US and the increased concurrence of IBD with HL or MS in identical patients. These findings lend additional support to the hypothesis that IBD may share a common risk factor with HL and MS. Additional epidemiologic studies will be needed to further delineate the risk factors that underlie the epidemiologic associations of CD and UC with HL and MS.
